# Primary carcinoid or sex cord-stromal tumor of the testis: A case report

**DOI:** 10.22088/cjim.14.1.147

**Published:** 2023

**Authors:** Ali Emadi Torghabeh, Mahmood Dolati, Masoumeh Gharib, Hamidreza Saghafi, Mohammadreza Saghafi

**Affiliations:** 1 Cancer Research Center, Mashhad University of Medical Sciences, Mashhad, Iran; 2 Department of Urology, Faculty of Medicine, Mashhad University of Medical Sciences, Mashhad, Iran; 3 Department of Pathology, Faculty of Medicine, Mashhad University of Medical Sciences, Mashhad, Iran; 4 Faculty of Medicine, Tehran Medical Branch of Islamic Azad University, Tehran, Iran; 5 Student Research Committee, Mashhad University of Medical Sciences, Mashhad, Iran

**Keywords:** Neuroendocrine tumor, Sex cord-stromal tumor of the testis, Testicular cancer Leydig, Sertoli, and granulosa cells, Testicular swelling

## Abstract

**Background:**

Carcinoid tumors of the testis are rare and orchiectomy is the preferred treatment. This type of testis tumors is rare and their differentiation from sex cord-stromal tumors is difficult.

**Case presentation:**

A 29‑year‑old man presented with right testicular mass and underwent radical orchidectomy. Histological examination showed neuroendocrine tumor, confirmed by immunohistochemistry and electron microscopy (Ethic code: IR.MUMS.REC.1400.237).

**Conclusion:**

Primary testicular neuroendocrine tumor is very rare. It is crucial to submit the entire gross specimen for histopathologic examination to rule out an existing of other germ cell elements. Our patient had a well-differentiated carcinoid tumor and after two years of follow-up (every three months), there was no recurrence or metastasis.

Carcinoid tumors are usually used to low to intermediate-grade neuroendocrine tumors (NETs) arising from the GI tract, lungs, or uncommon primary sites such as liver, gallbladder, kidneys, ovaries, and testes (1, 2). In the majority of cases, carcinoid tumors of the testis are primary tumors but they can be in kind of metastatic disease from the other sites. Their differentiation from sex cord-stromal tumors is difficult. Roughly 5 % of all testicular tumors are sex cord-stromal tumors. Testicular NETs origins are Leydig, Sertoli, and granulosa cells. The most common symptoms are testicular swelling mass or pain. They are often benign and metastatic disease is uncommon. Radical orchiectomy is the treatment of choice for both tumors (3-5). Neuroendocrine tumors (NETs) arise from the GI tract, lungs, or uncommon primary sites such as liver, gallbladder, kidneys, ovaries, and testes NETs of the testis are rare tumors that are often reported as a case report in studies. In this study, we report a case of a testicular tumor with this differential diagnosis.

## Case presentation

A twenty-nine-year-old man was referred to our hospital in October 2018 with a chief complaint of a painless mass in his right testis for three months(Ethic code: IR.MUMS.REC.1400.237). His medical history did not have any problems such as trauma or infection. His physical exam revealed a right painless and firm mass in the right scrotum. Ultrasonography showed a heterogeneous solid mass of 45 * 38mm with one calcified focus. Chest, abdomen, and pelvic CT scan and laboratory tests such as germ cell tumor markers were within the normal ranges. Therefore, he referred to a urologist for surgical management.

**Figure 1:**
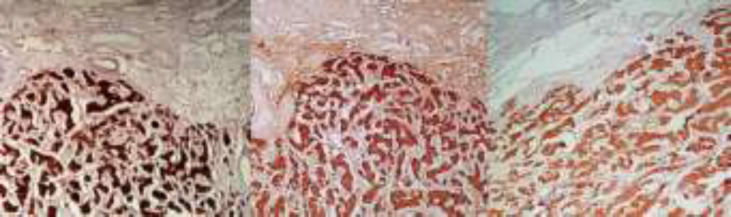
Well-circumscribed proliferation of solid nests and acini of monomorphic cells (H&E, 40*)

**Figure 2:**
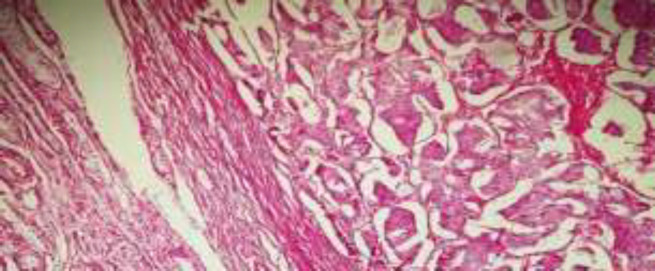
Positive immunohistochemical markers A) Ck, B) Chromogranin, C) Synaptophysin

After right radical orchiectomy, in pathologic laboratory, macroscopic examination showed a testis, measuring 6*6*3.5 cm with a well-delineated mass in the testis, measuring 4*4*3.3 cm, which had a solid, brown cut surface. Histopathologic evaluation showed a well-circumscribed proliferation of acini and solid nests of cells with prominent nucleoli and granular acidophilic cytoplasm. Mitosis was scarce and there were not any necrotic foci. To differentiate a sex cord-stromal tumor from a carcinoid tumor, immunohistochemical studies were performed. CK, chromogranin, and synaptophysin stained the tumoral cells, while vimentin, inhibin, calretinin, and CD30 were negative. Ki-67 marker stained 2% of cells. Morphologic and immunohistochemical (IHC) findings confirmed the diagnosis of a carcinoid tumor (figures 1-2). Postoperative evaluation with octreotide scan was normal and the patient followed-up without any adjuvant treatment.

## Discussion

The most common sites for carcinoid tumors are the gastrointestinal (GI) tract and pulmonary system, but they also occurred infrequently (near to 1 percent) in testes (6). Symptoms are dependent on involved sites such as bleeding or obstruction in the GI tract. The tumor may produce vasoactive products that can lead to carcinoid syndrome in 8 to 10 percent of the patients. The most common manifestations of this syndrome are diarrhea and flushing. The predominant cause of carcinoid syndrome is liver metastasis but it can see in locoregional tumors (7, 8). In the majority of cases, carcinoid tumors of the testis are primary tumors but they can be in kind of metastatic disease from the other sites. The most common symptom is the enlargement of the testis. Primary carcinoid tumors often originate from mature teratomas. This is notable that carcinoid tumors can occur in all ages.

Histopathologic features of sex cord-stromal tumor are not distinguishable from NET's of the testis, so immunohistochemical studies (IHC) should be performed. Histopathologically, there are two categories of differentiation: low-grade (well-differentiated) and intermediate-grade (moderately-differentiated) carcinoid tumors. Treatment, follow-up, and prognosis are based on these features.

Well-differentiated types have mitotic activity less than 2 per 10 HPF, with mild cellular atypia and marked nucleoli. Characteristics of moderately-differentiated carcinoid tumors are necrosis and moderate cellular atypia with mitotic rates more than 3 per 10 HPF and have metastatic potential. (9) MRI, CT scan, octreotide scintigraphy, and MIBG scan, urinary 5-hydroxy indol acetic acid(5-HIAA) measurement and IHC are frequently used for these purposes:

1. Looking for metastases, because there are no morphologic differences between primary and metastatic carcinoid tumors and also the correlation of them with the prognosis.

2. To differentiate from sex cord-stromal tumors and help oncologists rule them out and begin treatment. Carcinoid tumors of testes stain positive for serotonin, synaptophysin, chromogranin, substance P, and cytokeratin but stain negative for OCT4, CD30, CD117, TTF-1, and CDX-2 (10). Typically, in the first step, surgery (radical orchiectomy) should be done. Close follow-up after surgery seems to be a choice. Although additional adjuvant chemotherapy or radiotherapy are other options, they have a little proportion due to the unknown response of these tumors to such treatments and remain for the metastatic setting.

In Wang et al.’s study, 29 primary testicular carcinoid patients were included. The rate of metastasis was none in 20 cases with low-grade tumor and 1 in 4 cases with atypical features (11). Among the 10 patients enrolled in Reyes et al.’s study, all of them underwent orchiectomy. Nine patients were low grade.

After 4 years of follow-up, only 1 died with a moderately differentiated tumor. They concluded that the choice of treatment for low-grade tumors is orchiectomy with close follow-up but intermediate grade cases should receive additional therapy based on an individual clinical analysis (12). Our patient had a well-differentiated carcinoid tumor and after two years of follow-up (every three months), there was no recurrence or metastasis.

To differentiate teratoma from a carcinoid tumor, immunohistochemical studies were performed. CK, chromogranin, and synaptophysin stained the tumoral cells, while vimentin, inhibin, calretinin, and CD30 were negative. Ki-67 marker stained 2% of cells. Morphologic and immunohistochemical (IHC) findings confirm the diagnosis of a carcinoid tumor.
